# Building the capacity of older adults and community: findings from a developmental evaluation of United Way British Columbia’s social prescribing programs for older adults

**DOI:** 10.24095/hpcdp.44.9.04

**Published:** 2024-09

**Authors:** Laura Kadowaki, Bobbi Symes, Kahir Lalji, Grace Park, Wynona Giannasi, Jennifer Hystad, Elayne McIvor

**Affiliations:** 1 United Way British Columbia, Burnaby, British Columbia, Canada; 2 Fraser Health, Surrey, British Columbia, Canada; 3 Howegroup, Vancouver, British Columbia, Canada; 4 Catalyst Consulting, North Vancouver, British Columbia, Canada

**Keywords:** social prescribing, seniors, evaluation, healthy aging

## Abstract

**Introduction:**

Older adults with higher needs are ideal candidates for social prescribing interventions, given the complex and intersectoral nature of their needs. This article describes findings from a developmental evaluation of 19 social prescribing programs for older adults at risk of frailty.

**Methods:**

An evaluation of the programs was conducted from 2020 to 2023. We used data from three components of the evaluation: (1) initial evaluation data collected in 2020 and 2021; (2) program profiles developed in 2022; and (3) co-creation sessions conducted in 2023.

**Results:**

From startup until March 2023, the programs served a total of 2544 older adults. The community connectors identified factors at the individual, interpersonal, institutional, community and policy levels that contributed to the successful implementation and delivery of their programs (e.g. physician champions, communities of practice, strong pre-existing relationships with the health care system), as well as challenges (e.g. limited capacity of family physicians, lack of community resources). There was strong agreement among community connectors that successful social prescribing programs should include the following core elements: (1) making connections to needed community resources; (2) co-creation of a wellness plan with long-term clients or clients who require intensive supports; (3) ongoing follow-up and check-ins for clients with wellness plans; and (4) an assessment and triaging process for the prioritization of clients.

**Conclusion:**

To leverage the full potential of social prescribing interventions, it is essential that programs engage with a range of health and social care providers, that community connectors are skilled and well supported, and that adequate investments are made in the nonprofit and voluntary sector.

**Highlights**

- From a developmental evaluation of 19 social prescribing programs for older adults, we report on essential social prescribing program components, and facilitators and challenges of program implementation and delivery.
- The key challenge reported by programs was engaging with family physicians, suggesting that programs can benefit from having physician champions and engaging with a broad range of health and social care providers to make referrals.
- The community connector position emerged as essential to the success of the social prescribing interventions.
- A strong nonprofit and voluntary sector is required to leverage the full potential of social prescribing.

## Introduction

Social prescribing is a health promotion intervention designed to connect individuals with community resources to address their nonmedical needs. The intervention is considered to have originated in the United Kingdom, and examples of programs can be found across Europe, Asia, North America and Australia.^1^ Social prescribing builds on current global health trends and priorities, including integrated care and care coordination, person-centred care, co-design and co-production, strengths-based approaches, asset-based community development, health promotion, self-determination theory and quadruple aim.^1^

There are two common pathways through which social prescribing interventions may take place: (1) direct referrals by a primary care provider to needed community resources (e.g. arts programs, exercise, nature) or (2) a referral to a community connector who works with the individual to identify and address unmet needs.^2^ We use the term “community connector” throughout this article because it is the terminology used in British Columbia (BC), but these individuals are also commonly known as “link workers” or “navigators.” 

Social prescribing interventions using a community connector usually consists of three stages: (1) a primary care provider refers the individual to a community connector; (2) the community connector works with the individual to identify their needs and refer them to appropriate community resources; and (3) the individual engages with new community resources or activities.^3^ Traditionally in social prescribing models, primary care providers are the source of referrals to community connectors; however, some models now target a wide range of health and social care providers for referrals.^3^,^4^

Older adults with higher care needs—such as those experiencing frailty, multiple chronic conditions, loneliness or poor nutrition—are ideal candidates for social prescribing interventions, given the often complex and intersectoral nature of their needs. In Canada, it has been estimated that over half of older adults are either frail (22%) or pre-frail (32%).^5^ About one in five older adults also lacks social support.^6^ Furthermore, between 17% and 33% of older Canadians are lonely at least some of the time, depending on age and gender (the prevalence of loneliness increased to 26%–42% during the pandemic).^7^

Two recent systematic reviews illustrate the current state of knowledge about social prescribing programs for older adults.^3^,^8^ In the systematic review by Percival et al.,^3^ seven articles were identified that met their inclusion criteria (i.e. a social prescribing intervention for older adults with quantitative outcome data). The social prescribing programs described in the articles relied on a range of health and social care providers for referrals, and community connectors referred clients to a variety of community resources (e.g. art programs, health promotion classes, social activities). The studies in the review most commonly reported on psychosocial outcomes, and consistently found improvements on mental well-being measures. Positive impacts were also observed for physical health outcomes in two studies. However, the findings on health care utilization were mixed. 

A second, complementary systematic review was conducted by Grover et al.^8^ and identified eight qualitative studies that met their inclusion criteria (i.e. studies of the experience, outcomes or processes of social prescribing programs from the perspective of older adults or service providers). Using a meta-aggregation approach, the authors synthesized the results into five findings: (1) personalized experiences (i.e. the need for person-centred approaches to support older adults living with chronic conditions); (2) providers and connectors (i.e. role of the general practitioners and community connectors in making older adults feel supported); (3) behaviour change (i.e. studies reported on successful motivators and behaviour change techniques such as increased self-confidence and building skills for long-term self-management); (4) environment (i.e. familiar and well-chosen places for activities contributed to the positive engagement of participants); and (5) outcomes (i.e. most of the articles reported on positive outcomes for older adults related to health, lifestyle and/or socialization).

To help build our knowledge on social prescribing programs for older adults within the Canadian context, in this article we describe findings from a developmental evaluation of 19 social prescribing programs for older adults at risk of frailty. These social prescribing programs were being implemented as a part of a series of demonstration projects called “Integrated Community-Based Programs for Older Adults with Higher Needs” that were funded by the Province of British Columbia. United Way British Columbia (United Way BC) was the backbone organization for the social prescribing programs, which were being implemented in 19 communities across BC by local community-based seniors’ services. Since the completion of the successful demonstration project, United Way BC has been undertaking a phased approach to roll out social prescribing programs across the province, funded by the Province of BC, with the goal of having a community connector in placein each local health area by 2025/26. 

## Methods

This article reports on the findings of a developmental evaluation of the social prescribing programs conducted over the period 2020 to 2023 by an externally contracted group (Howegroup). As this was a program evaluation, it did not fall under the scope of research ethics board review. 

***Overview of social prescribing interventions***

Using the Template for Intervention Description and Replication(TIDieR) checklist as a guide,^9^ Table 1 describes key characteristics of the social prescribing interventions.

**Table 1:** Description of 19 social prescribing programs for frail older adults, British Columbia, 2020 to 2023

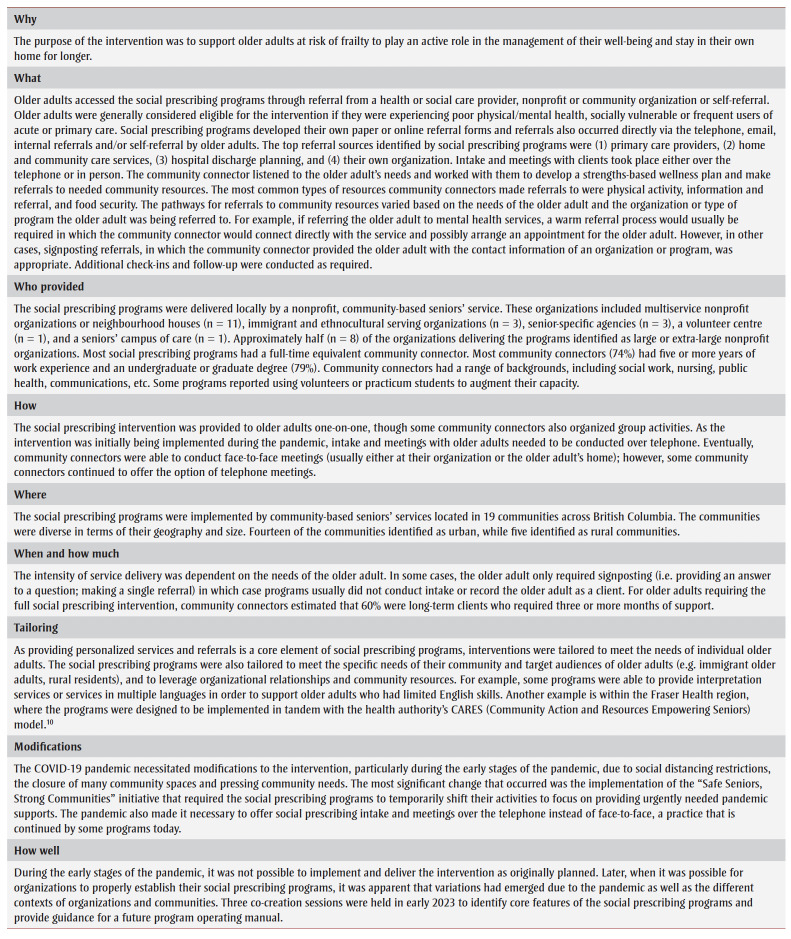

***Evaluation methods***

Originally, the intention was to conduct both developmental and summative evaluations of the programs using a mixed methods approach. However, the COVID-19 pandemic resulted in significant disruptions to the implementation of the social prescribing programs and the planned collection of longitudinal outcome data from program clients (i.e. delays in program start-up, challenges engaging with older adults, alteration of intended program activities, insufficient time for follow-up). While baseline outcome data were collected from an initial 504 clients, the evaluation was only able to obtain follow-up responses from 34 clients at the six-month follow-up. This resulted in significant data validity concerns. Further time and evaluation are required to determine the outcomes for individual older adults participating. As a result, this article focusses on the findings from the developmental evaluation components. 

We draw on data that were collected from the programs’ community connectors via three components of the evaluation: (1)initial evaluation data collected from community connectors in 2020 and 2021; (2) programs profiles developed in 2022; and (3) co-creation sessions conducted in 2023. Invitations were sent out by United Way BC asking the community connectors (n=19) to participate in the various evaluation components. Each of these components are described in greater detail below. 

After the pandemic began, an online COVID-19 check-in survey (n=19) was conducted with program staff to determine how the social prescribing programs were being impacted. The survey included closed-ended questions about the extent to which the programs had shifted or been offered as planned due to the pandemic and whether the intake process had started. Participants also could provide additional written feedback via the survey. A focus group was also held with the community connectors in 2021 (n=10) to explore the program implementation in more detail. Discussion questions were posed by the evaluators on program shifts due to the pandemic, service delivery strategies, and feedback on available support and suggestions for program improvements. 

In 2022, after the social prescribing programs had been able to fully resume their regular activities, data were collected on each program from telephone interviews (n=19) and an online survey (n=18). The survey was used to collect basic information on the programs, including characteristics of the organization delivering the program (e.g. size, location), staffing information (e.g. education and experience of community connector) and referral sources (e.g. main sources of referrals to and from programs). The semistructured interviews were used to collect information on approaches to social prescribing, facilitators and limiting factors, areas requiring further support, successes and challenges, partnerships, and lessons learned. 

The data from the interview and survey for each individual program were then combined into a program profile to provide a fulsome picture of the organization’s social prescribing program characteristics, approach, and successes and challenges. The program profiles were collectively analyzed to identify program challenges and successes and potential program guidelines and best practices. Key findings from the program profiles were presented back to the community connectors at a community of practice meeting for validation and refinement. Due to staff turnover, one social prescribing program was able to provide only limited program profile data. A second program was unable to complete the online survey. 

Finally, three co-creation focus group sessions were conducted with the community connectors in early 2023. Two of the sessions were conducted in person (with a virtual option for those unable to attend in person) and one was conducted fully online. The sessions built on the data collected via the program profiles and were held to develop consensus on social prescribing program design and delivery components, as well as to identify areas for future support. Feedback was collected from the community connectors via group discussions and supplemental polls, and the notes from the sessions were examined to identify key findings. The co-creation sessions were semistructured, with discussion prompts and questions posed by the facilitators (e.g. what are core vs. optional program elements; share examples of successful relationship-building in your community; possible community of practice formats and topics for meetings, etc.), but also provided flexibility for the discussions to evolve organically. Feedback was also collected from the community connectors via close-ended polls. The notes from the sessions were compiled into summaries for each session. 

Discussions at co-creation session 1 focussed on the characteristics of program clients who benefit most, core program elements and desired training opportunities. Discussions at co-creation session 2 included further discussion and polls on the characteristics of program clients who benefit most and core program elements, as well as discussion of strategies to support program referrals. Discussions at co-creation session 3 focussed on the role of networks, communities of practice and strategies to support program referrals.

In this paper, we use the evaluation data from 2020 and 2021 to provide brief background context for the implementation process and challenges that occurred due to the pandemic. The primary focus of the paper is the program profile and co-creation session data that provide insights into the social prescribing program implementation and delivery when normal operations began to resume. Thematic analysis was used to identify themes and key findings from the program profiles and co-creation session. We use the social-ecological model (described in the next section) to structure these findings in the paper.

***Social-ecological model***

The social-ecological model has its origin in the work of Bronfenbrenner on human development.^11^ Concern about individualistic approaches to health promotion interventions led researchers to examine the general ecological model as a model for health promotion. McLeroy et al.^12^ developed a variation of Bronfenbrenner’s model; theirs is referred to as the social-ecological model. The social-ecological model proposes a nested model consisting of five levels: intrapersonal (i.e. characteristics of the individual); interpersonal (i.e. formal and informal social support networks and systems); institutional (i.e. processes, norms, rules and regulations of institutions); community (i.e. relationships among organizations, institutions and networks); and public policy (i.e. laws and policies).^12^ Compared to earlier ecological models, the social-ecological model more explicitly acknowledges the social environment, institutions and cultural contexts that influence the implementation of health promotion interventions and shape the health and well-being of individuals.^13^

## Results

In the first section of the results, we provide an overview of the implementation of the social prescribing programs, including the impacts of the COVID-19 pandemic. In the second section of the results, based on the program profile and co-creation session data, we describe key factors at the five levels of the social-ecological model that influenced the implementation and delivery of the intervention. The “Who provided” section of Table 1 shows basic information on the community connectors who participated in the evaluation and their organizations.

***Implementation of the social prescribing programs***

The social prescribing programs were slated to be implemented on a rolling basis between summer 2019 and summer 2020. However, the COVID-19 pandemic caused significant disruption to the start-up of the programs due to closure of organizational locations, inability to meet face-to-face with older adults, reduced referral opportunities and changing support needs of older adults. In a check-in survey of the social prescribing programs conducted in fall 2020, only three programs reported they were offering services as planned or more effectively than originally planned; five programs reported they had not even been able to start offering social prescribing services. 

While the programs had very limited success offering actual social prescribing services during their first year of operation, they played an important role in offering COVID-19 supports to vulnerable older adults. In partnership with the BC Ministry of Health, the Office of the Seniors Advocate and 211 British Columbia (a province-wide information and referral service), United Way BC coordinated a province-wide response to the pandemic called “Safe Seniors, Strong Communities.” Beginning in March 2020, the social prescribing programs, as well as other United Way BC Healthy Aging–funded initiatives, shifted their programming to focus on providing pandemic supports. As of March 2023, Safe Seniors, Strong Communities has provided 1 294 248 services (i.e. check-ins, grocery shopping and delivery, prepared meal delivery, prescription pick-up and drop-off, etc.) to 39220 older adults.

Most of the social prescribing programs did not resume their intended social prescribing operations until 2021. During the first phase of the demonstration project (from the time the programs initially started up until March 2022), when the impacts of the COVID-19 pandemic were the most intense and programs were in the process of establishing themselves or resuming regular activities, the programs served a total of 1110 unique clients (average of 58 per program). Most of these clients were served in 2021/22 due to pandemic disruptions. In the second phase of the demonstration project (April 2022 to March 2023), when the programs were fully established, a total of 1434unique clients were served (average of 75 per program). 

Table 1 describes the approaches and implementation of the social prescribing programs. During the co-creation sessions, the core elements of social prescribing programs for frail older adults were discussed. In polls conducted at the session, there was consensus among most of the community connectors that the following four activities should be core program elements: (1) making connections to needed community resources for the older adult (100% agreement); (2) co-creating a wellness plan for clients requiring more intensive supports (i.e. long-term support lasting more than three months or intensive one-on-one short-term support; 74% agreement); (3) providing ongoing follow-up and check-ins for clients with wellness plans (74% agreement); and (4) establishing an assessment and triaging process for prioritizing clients if referrals exceed program capacity (68% agreement). Community connectors were ambivalent as to whether two additional activities should be core program elements: (1) assistance with health system navigation (58% agreement); and (2) reporting back to referring health care providers (32% agreement). 

***Facilitators and challenges of program implementation and delivery***

Based on the data from the program profiles and co-creation sessions, we describe in the sections below key facilitators and challenges that influenced program implementation and delivery at the five levels of the social-ecological model. It is important to note that many of these factors are cross-cutting and span multiple levels of the model; therefore, while we have chosen to discuss them at a specific social-ecological level, most intersect with additional levels of the model. While there were a number of challenges that occurred specifically due to the pandemic, we have focussed on those challenges that would be relevant for a wide range of contexts. 

**Individual level**

During the co-creation session discussions and polls, there was unanimous (100%) agreement among the community connectors that individuals with limited family and social support benefit the most from social prescribing. Additionally, the intervention was also deemed to be most beneficial for older adults who desire support and are motivated to participate (84% agreement) and older adults who are able to set goals and engage over time (84% agreement). 

**Interpersonal level**

At the interpersonal level, relationships between the community connector and the older adults, health care system and other community resources emerged as essential. From the program profiles it was apparent that the experience, community knowledge and relationships of community connectors were key to the success of the programs. Community connectors reported leveraging their knowledge and pre-existing relationships with other nonprofit and health care organizations in their community in order to (1) offer their clients referrals to needed community resources; (2) address service gaps and develop new activities and services to meet the needs of older adults (e.g. educational talks, digital technology training, interpretation services); and (3) share information and resources to better support clients. A common challenge for the programs that struggled with getting their social prescribing service off the ground was inexperienced community connectors or staff turnover. In the co-creation sessions, the most recommended training topics to enhance community connector skills were trainings on how the health care system works, available health care and community resources, identifying mental health crises, motivational interviewing and boundary setting. 

***Institutional level***

At the institutional level, the community connectors identified current and desired supports from United Way BC to support the social prescribing programs. Most community connectors identified in the program profiles the value of having a community of practice so they could connect with and learn from the challenges and successes of the other social prescribing programs. A formal community of practice exists for all programs and Fraser Health also operates a community of practice for the programs in their region (individuals from programs outside of the region are also able to attend some of their sessions that are not specific to Fraser Health). In the co-creation sessions, most community connectors voiced their preference that community of practice meetings occur monthly or bi-monthly, be chaired by a content expert and include regular open discussion time in addition to structured presentations and activities. 

During the program profile interviews and co-creation sessions, community connectors also identified improved marketing and communication supports from United Way BC as potential facilitators for increasing referrals and strengthening programs’ credibility. In the co-creation sessions, community connectors emphasized the importance of communicating to potential clients that social prescribing programs offer services that are person-centred and strengths-based, and can help to enhance the independence and social connections of older adults. Furthermore, it is important to clearly explain the role of the community connector and types of support that can be offered. 

When communicating with health care providers, community connectors emphasized clearly explaining what the program is (e.g. connects older adults to needed community resources), who the target audience is (e.g. older adults who need social connections and supports, underserved older adults) and the benefits of the program to older adults (e.g. alleviating loneliness, increasing quality of life and well-being, improving skills and confidence). The importance of establishing the credibility of social prescribing programs was emphasized by, for example, stating that these programs are funded by the Ministry of Health and affiliated with United Way BC. Community connectors also suggested developing a brochure that health care providers can give to an older adult during their appointment. 

***Community level***

The main challenge for programs that emerged from the program profile data was building relationships with and getting referrals from the health care system (specifically family physicians). Often there was a lack of understanding among health care providers of what social prescribing was and who would be appropriate to refer to the programs. Some community connectors reported feeling they were not taken seriously when they tried to conduct outreach to family physicians’ offices. While originally it was intended that the social prescribing programs would primarily target family physicians for referrals, in response to the challenges that were encountered, most programs pivoted to outreach to a wider range of health care providers. Often programs reported having more success conducting outreach to home health teams, community health centres, older adult mental health teams and hospital discharge teams. 

From the program profile data, it was also apparent that the strength of social prescribing programs’ relationships with the health care system varied significantly. Having a physician champion or a pre-existing, close working relationship with the health care system assisted some programs in getting buy-in and referrals from health care providers. 

Generally, social prescribing programs within the Fraser Health region were the most successful at building relationships with health care providers, as they were implemented with the support of a physician champion as a part of the health authority’s Community Action and Resources Empowering Seniors (CARES) model. Community connectors from this region commented on how the physician champion was able to provide them with credibility and open doors for them (some community connectors from outside of this health region even commented on how the physician champion was able to offer advice or help make connections for them). 

Several Fraser Health programs were also working with health care partners to pilot social prescribing in assisted living facilities or acute care settings. Outside of the Fraser Health region, programs generally reported making progress building relationships with health care providers, but this was often slow, and it took more time to build relationships. 

***Policy level***

Family physician shortages acted as a barrier to the intervention, as several community connectors observed that family physicians were overworked and lacked the capacity to engage with the social prescribing programs. It was also highlighted, particularly in the rural context, that a notable number of older adults do not have a family physician. For example, in a small rural community it was reported that one in five individuals did not have a family physician and for those with a family physician wait times were six or more weeks.

Despite the significant efforts of community connectors to make referrals for their clients, lack of community resources to which to refer clients emerged as a commonly reported challenge. This was particularly a concern in rural communities, with some community connectors suggesting social prescribing programs need the flexibility to create activities and services to fill gaps, in addition to pushing referrals. During the pandemic, the issue of the availability of community resources was intensified by the closure of many organizations and community spaces. While there were opportunities to refer older adults to online activities, not everyone has access to digital technology or sufficient digital literacy to make use of these opportunities. Furthermore, community connectors identified that even after more organizations and locations began to open up and offer in-person activities, some older adults remained hesitant to engage in person. 

Some vulnerable older adults require significant social and emotional support to help them engage with community resources (e.g. providing transportation, accompanying them on outings into the community, interpretation), which can challenge the capacity of social prescribing programs. Several community connectors commented on the need to maintain boundaries and resist trying to fix problems that are outside of the scope of their program. Community connectors also identified that some clients have complex needs and require referrals for services that are not available or are at capacity in their community (e.g. social housing, mental health services, food security). While community connectors recognize they cannot solve all the problems of their clients, it is troubling to them when their clients have serious health-, housing- or poverty-related concerns they are unable to address. 

## Discussion

The findings from our developmental evaluation highlight the importance of planning for the implementation and delivery of social prescribing interventions at all levels of the social-ecological model. In particular, our study highlights the impact that higher level institutional-, community- and policy-level factors can have on the implementation of social prescribing programs. 

The key challenge reported by community connectors was building relationships with family physicians to facilitate referrals, a challenge that spans the interpersonal, institutional, community and policy levels of the social-ecological model. Challenges receiving referrals from family physicians have similarly been reported in other studies due to lack of understanding of social prescribing programs and the lack of family physicians’ time.^8^,^14^ Indeed, gaining buy-in from family physicians and legitimizing social prescribing programs in the eyes of the health care system has been identified as a key step in successful social prescribing program implementation.^15^ The most impactful strategy that emerged from our study for addressing this issue was cultivating a physician champion, a strategy that has also been identified in other research.^14^ Additional recommended strategies from the literature to encourage the engagement of family physicians include regular education and information sessions, providing feedback letters on referrals, embedding social prescribers in physicians’ offices and ensuring the referral process is brief and easy.^14^,^15^

Contrary to the literature, in our study when community connectors were polled, the majority did not identify reporting back to the referrer as a core program element. This perhaps is reflective of the context in BC and the perception that family physicians are overburdened and lack the capacity or interest to review such documents. A 2022 poll conducted by Angus Reid reported that 59% of British Columbians lack access to or find it difficult to access a family physician,^16^ supporting that there is a need for social prescribing programs to engage with a broader range of health care providers. Many social prescribing programs reported greater success connecting with other health care providers (e.g. home and community care, mental health teams, hospital discharge teams) who may have a greater capacity for engagement due to the presence of team members such as case managers to facilitate referrals. 

At the other end of the social prescribing process, some community connectors reported a lack of appropriate organizations and services to refer older adults to, representing a key community- and policy-level challenge. The need for a strong nonprofit and voluntary service sector to support social prescribing programs has previously been reported in the literature.^14^,^17^-^19^ Hamilton-West et al.^17^ caution there is the potential that social prescribing will increase the strain on nonprofit and voluntary services that are already struggling with capacity and downloading of responsibility from the health and social care systems. 

As the social prescribing programs in BC were implemented during the COVID-19 pandemic, it is unclear to what extent gaps in community resources may have been due to pandemic closures versus inadequate capacity and investment in the nonprofit and voluntary sector. The agreement (68%) among community connectors that an assessment and triaging process for referrals should be a core program element indicates there are capacity concerns beyond the context of the pandemic. Furthermore, some community connectors reported older adults having significant unmet needs beyond the capacity of community resources to address, suggesting that gaps in capacity exist more broadly in major sectors such as health and housing. In rural communities, these gaps were observed to be especially acute. 

At the interpersonal and community levels, the community connector role emerged as essential to the success of the social prescribing interventions. Programs with experienced community connectors who were knowledgeable about community assets and had pre-existing relationships with other organizations and providers in the community generally reported more success receiving and making referrals. 

Furthermore, some community connectors reported going beyond just making referrals to offering additional supports for accessing resources (e.g. arranging transportation, accompanying on outings) and organizing or developing new community resources or activities to meet the needs of older adults in their community. This finding suggests that there is the potential for community connectors to play a broader role in supporting vulnerable older adults and contributing to capacity-building within their community. However, it also raises the possibility that community connectors might become overburdened if they are not adequately resourced and supported. In the United Kingdom, social prescribing approaches that incorporate community-asset building are being developed.^20^,^21^ For example, in Rotherham, grant funding is available for both the social prescribing referral processes and the activities and programs to which the clients are being referred.^20^

At the institutional level, an important finding in our evaluation was the value of communities of practice and how community connectors benefit from the opportunity to share and engage in discussions with other community connectors. Community connectors in the United Kingdom have reported the benefits of shadowing others,^22^ suggesting that one-on-one mentorship or support may also be beneficial for less experienced community connectors. Furthermore, previous research has warned about the potential stress and burnout that can occur when dealing with high-needs and complex clients;^15^,^22^,^23^ therefore it is important that community connectors receive effective training and emotional supports. Tierney et al.^15^ also note that when staff turnover occurs in the community connector position, it can take time to rebuild the community knowledge and relationships that were held by the previous individual. 

***Strengths and limitations***

A key strength of this evaluation was the large number of social prescribing programs involved (n=19), including programs implemented in rural communities. A second strength of the research was the multiple data collection points that spanned 2020 to 2023, which allowed for a more fulsome picture of the intervention to be developed. Furthermore, the evaluation adds to our knowledge on social prescribing programs for frail older adults, a vulnerable target population on which there has been only limited Canadian social prescribing research to-date. 

There are several limitations of the research that should also be noted. First, due to the disruptions caused by the COVID-19 pandemic, it was not possible to evaluate the impacts of the social prescribing interventions on individual older adults. Second, due to staffing challenges, data collection from two programs for the program profiles were incomplete or limited. Third, as our data were collected in the province of BC primarily during the COVID-19 pandemic, some of the findings may not be generalizable to other contexts.

## Conclusion

As populations around the globe age, there will be increased interest in interventions to support the nonmedical needs of frail older adults and older adults who are isolated or lack social supports. The developmental evaluation findings reported on in this article contribute to our understanding of social prescribing programs for older adults at multiple levels of the social-ecological model, including essential program components and facilitators, and challenges of program implementation and delivery. Key lessons that have emerged from this research include the benefits of social prescribing programs engaging with a broad range of health and social care providers who can make referrals, beyond just family physicians; the value of physician champions and communities of practice; the essential knowledge mobilization, capacity-building and relationship-building role of the community connector; and the importance of adequately investing in nonprofit and voluntary sectors in order to leverage the full potential of social prescribing.

## Acknowledgements

We would like to thank the 19 community-based seniors’ services who are delivering the social prescribing programs and the community connectors for their participation in the evaluation. 

We would like to acknowledge the Integrated Community-Based Programs for Older Adults with Higher Needs are funded by the Province of British Columbia

## Conflicts of interest

Laura Kadowaki, Bobbi Symes and Kahir Lalji are employed by United Way British Columbia. Grace Park is a contracted regional medical director for community health services in Fraser Health. Wynona Giannasi, Jennifer Hystad and Elayne McIvor are independent consultants from the Howegroup who were contracted to conduct the evaluation of the social prescribing programs.

## Authors’ contributions and statement

LK, BS, KL, GP, WG, JH, EM: conceptualization.

WG, JH, EM: investigation, methodology, project administration.

WG, JH, EM, LK: formal analysis.

LK: writing—original draft.

LK, BS, KL, GP, WG, JH, EM: writing—review and editing.

The content and views expressed in this article are those of the authors and do not necessarily reflect those of the Government of Canada.
